# Supplementary material for: Modifying the Expression of Cysteine Protease Gene PCP Affects Pollen Development, Germination and Plant Drought Tolerance in Maize
Source: Int J Mol Sci. 2023 Apr 17;24(8):7406. doi: 10.3390/ijms24087406 (PMC10138719; doi:10.3390/ijms24087406)
Supplement: Supplementary file 1 [file ijms-24-07406-s001.zip › ijms-2307821-supplementary.pdf]

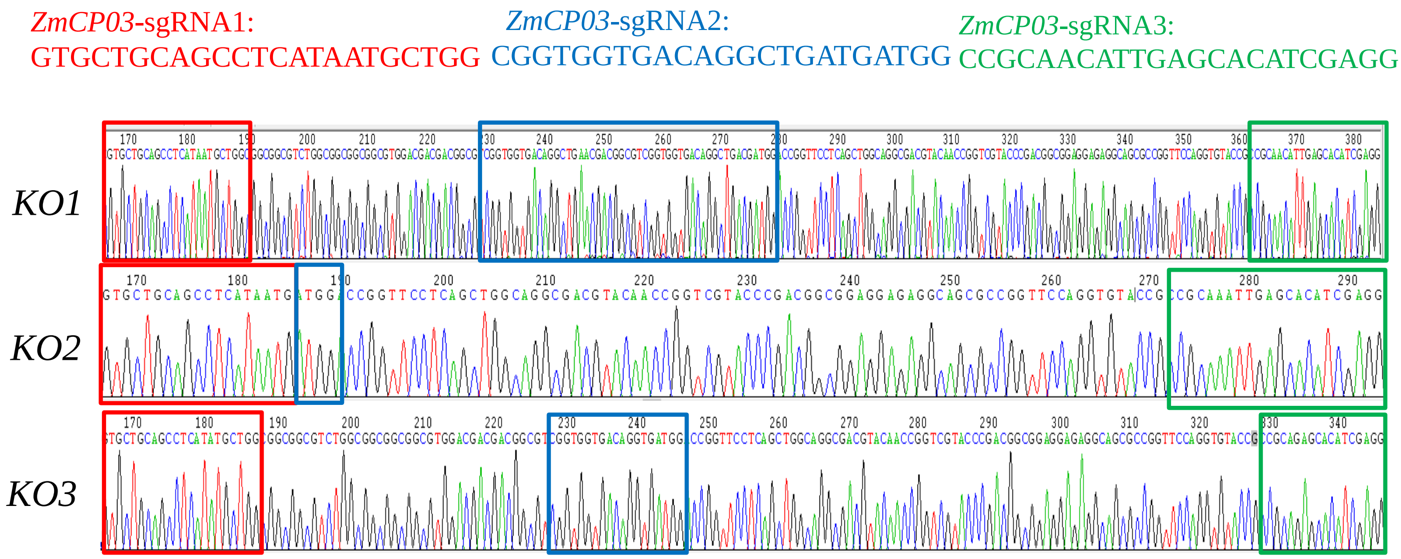

Figure S1. The sequencing results of pollen grains of T2 mutants.

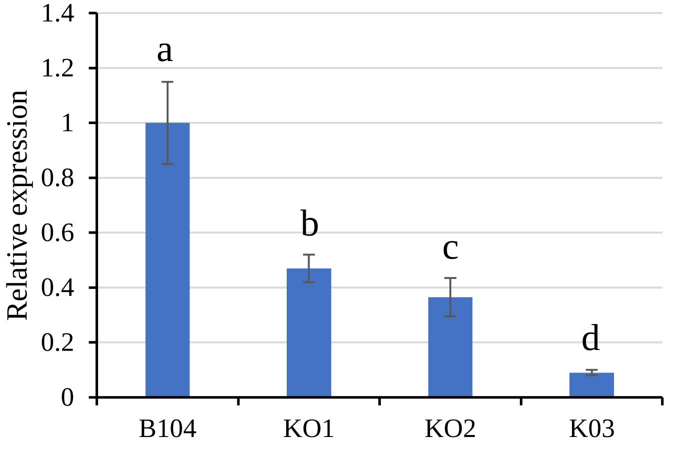

Figure S2. The relative expression levels of *PCP* were analyzed via RT-qPCR in *pcp* mutant lines. Data represent means ± SD of three replicates. Significant differences were indicated with different letters ($P < 0.05$, one-way ANOVA).

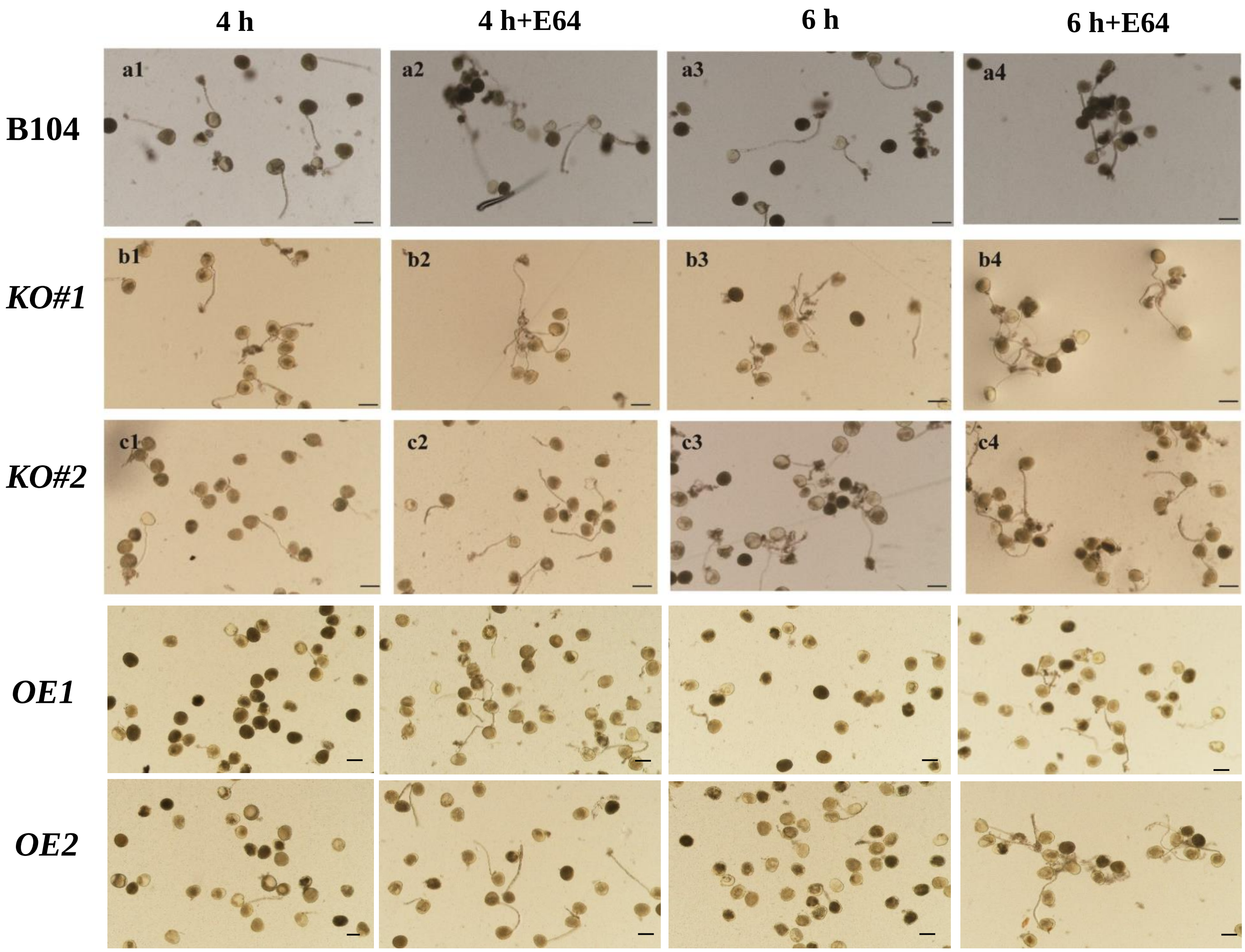

Figure S3. Pictures of pollen germination and growth *in vitro* between wild type and transgenic lines after 4 h and 6 h incubation. Scale bar: 100 µm.

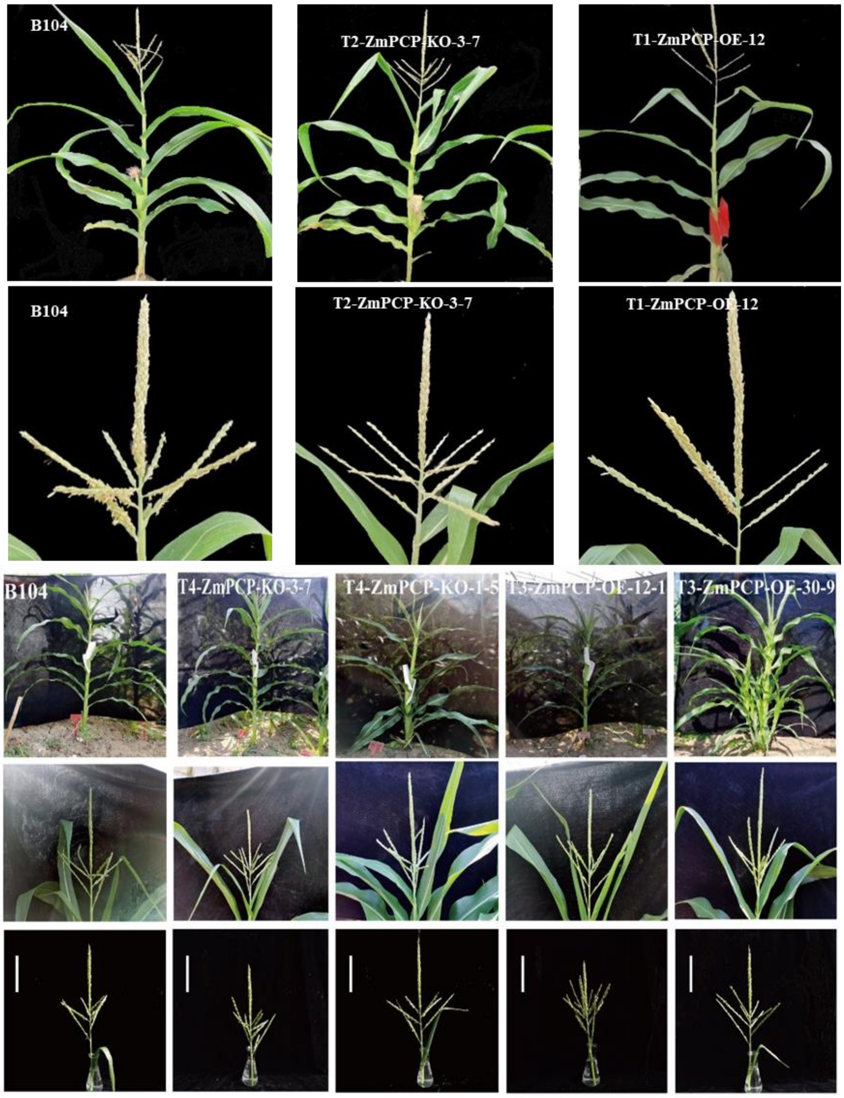

Figure S4. Phenotypic observation of WT and transgenic maize at flowering stage. Scale bar: 10 cm.

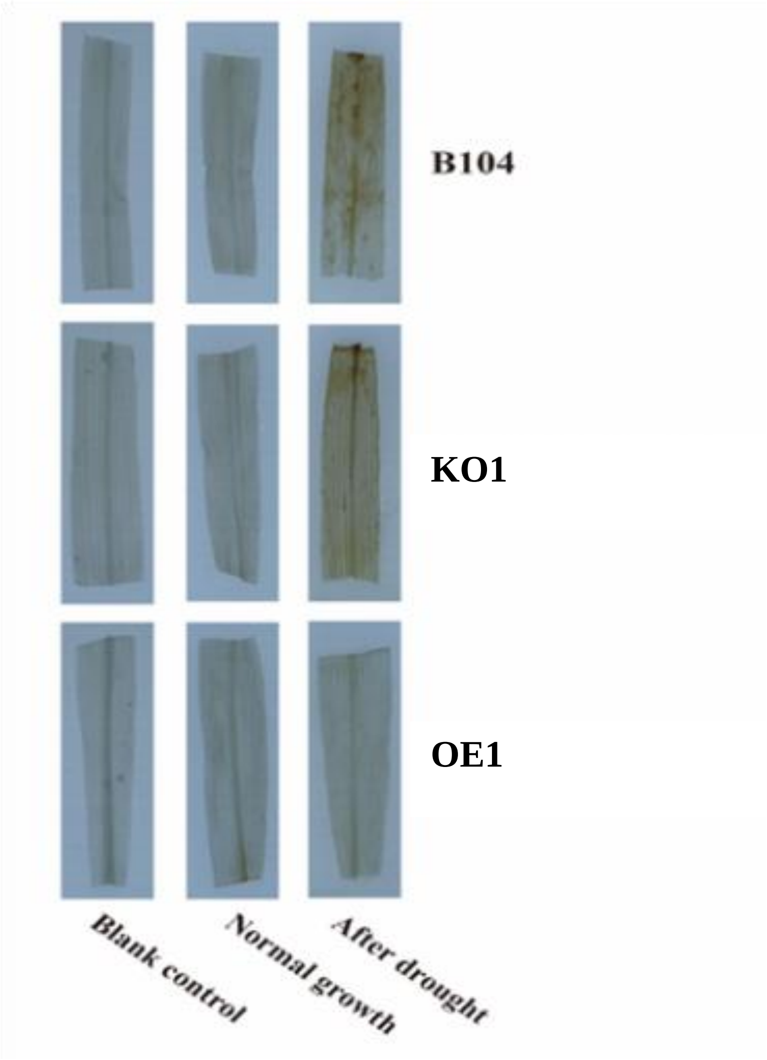

Figure S5. Measurement of H_2O_2 content in WT and transgenic maize in response to drought stress by DAB staining.

Table S1. PCR primers used in this study.

Primer name	Primer sequence (5'-3')	Usage
sgRNA-T1-F	cagt ggtctca GGCA CCTCGATGTGCTCAATGTTG	Synthesis of sgRNA
sgRNA-T1-R	cagt ggtctca AAACCAACATTGAGCACATCGAGG	
sgRNA-T2-F	cagt ggtctca GCCG TGCTGCAGCCTCATAATGC	
sgRNA-T2-R	cagt ggtctca AAAC GCATTATGAGGCTGCAGCA	
sgRNA-T3-F	cagtggtctcaTGTGCGGTGGTGACAGGCTGATGA	
sgRNA-T3-R	cagt ggtctca AAACTCATCAGCCTGTCACCACCG	
psgA-T1	GACCATAGCACAAGACAGGCGT	Detection of intermediate vectors
psgB-T2	CGAATGAGCCCTGAAGTCTGAAC	
psgC-T3	CATTTCATTACCTCTTTCTCC	
pOSCas9-ZmCP03-F	GATGGGTTTTTATGATTAGAGTCC	Detection of expression vectors
pOSCas9-ZmCP03-R	GGCTCGTATGTTGTGTGG	
